# An escalating continuum of learning and attention difficulties from premutation to full mutation in female carriers of *FMR1* expansion

**DOI:** 10.3389/fneur.2023.1135630

**Published:** 2023-05-02

**Authors:** Lidia V. Gabis, Meirav Shaham, Odelia Leon Attia, Tamar Kowal, Sivan David, Yonit Banet-Levi, Shahar Shefer, Daniel Gabis, Dana Mula-Topf, Michal Avrech Bar, Orit Bart, Osnat Segal

**Affiliations:** ^1^Sackler Faculty of Medicine, Tel Aviv University, Tel Aviv-Yafo, Israel; ^2^Maccabi Healthcare, Tel Aviv-Yafo, Israel; ^3^Keshet Autism Center Maccabi-Wolfson, Holon, Israel; ^4^Department of Statistics, University of Haifa, Haifa, Israel; ^5^Department of Communication Disorders, Sackler Faculty of Medicine, Tel Aviv University, Tel Aviv-Yafo, Israel; ^6^Department of Occupational Therapy, School of Health Professions, Sackler Faculty of Medicine, Tel Aviv University, Tel Aviv-Yafo, Israel; ^7^Weinberg Child Development Center at Safra Children's Hospital, Sheba Medical Center, Ramat Gan, Israel; ^8^College of Management, Rishon LeZion, Israel; ^9^Tel Aviv Sourasky Medical Center Ichilov, Tel Aviv-Yafo, Israel

**Keywords:** Fragile X, FXS, *FMR1*, Fragile X carriers, learning disabilities (LD), attention deficit and hyperactivity disorder (ADHD), premutation

## Abstract

**Objective:**

Carriers of Fragile X premutation may have associated medical comorbidities, such as Fragile X-associated tremor and ataxia (FXTAS) and Fragile X-associated premature ovarian insufficiency (FXPOI). We examined the Fragile X premutation effect on cognition, and we assumed that there is a direct correlation between the continuous spectrum of specific learning and attention deficits to the number of CGG repeats on the *FMR1* gene.

**Methods:**

A total of 108 women were referred to our center due to a related Fragile X syndrome (FXS) patient, 79 women carried a premutation of 56–199 repeats, and 19 women carried a full mutation of more than 200 CGG repeats on *FMR1* gene. Genetic results of CGG repeats, demographic information, structured questionnaires for ADHD, learning disabilities of language and mathematics, and independence level were analyzed in women carrying the *FMR1* premutation and compared to the group carrying the full mutation. Women with FXS and FXTAS were excluded.

**Results:**

When analyzed as a continuum, there was a significant increase in the following complaints which were associated with a higher number of repeats: specific daily function skills such as driving a car, writing checks, disorientation in directions, and also specific learning difficulties such as spelling and math difficulties. Additionally, when tested as a categorical independent variable, we observe that women with the full mutation were more likely to have ADHD or other learning disability diagnoses in the past than during premutation (<200 CGG repetitions).

**Conclusion:**

Specific learning and attention difficulties and resulting daily function difficulties correlate with an increased number of CGG repeats and are more likely to be associated as a common feature of premutation and full mutation in a female premutation carrier. Despite evidence of learning and attention difficulties, it is encouraging that most female carriers of the premutation and full mutation function well in most areas. Nevertheless, they face significant difficulties in specific areas of functioning such as driving, and confusion in times and schedules. Those daily function skills are mostly impacted by dyscalculia, right and left disorientation, and attention difficulties. This may aid to design specific interventions to address specific learning deficits in order to improve daily function skills and quality of life.

## Introduction

Fragile X syndrome (FXS) is the most prevalent genetically inherited cause of autism and intellectual disability. Carriers of Fragile X premutation are quite prevalent [1:259 worldwide] and twice as prevalent in Israel [1:140] (1).

Fragile X syndrome is caused by an abnormality in the Fragile X messenger ribonucleoprotein 1 (*FMR1*) gene. *FMR1* gene is located on the X chromosome at Xq27.3 and is normally comprised of 40 or fewer cytosine–guanine–guanine (CGG) repeats. The number of CGG repeats defines normal allele, premutation (“carriers”), and full mutation (2).

The Fragile X premutation is characterized by a large CGG repeat track (55–199 repeats) in the 5'UTR of the *FMR1* gene. In previous studies, the phenotype of carrier status has been rendered asymptomatic, and its main significance has been limited to the risk of further expansion to a full mutation in the offspring of carriers. More recent studies reveal that premutation alleles of the *FMR1* gene are associated with several medical conditions (1, 3, 4), and the two most studied topics are Fragile X-associated tremor/ataxia syndrome (FXTAS) and Fragile X-associated primary ovarian insufficiency (FXPOI) (5). The main clinical presentations of premutated individuals with FXTAS are gait, memory, and executive dysfunction. This syndrome has been reported in 40% of men and 8% of women who are older than 50 years, carrying the premutation (6, 7).

Primary ovarian insufficiency (POI) is a condition considered to be present when a woman younger than 40 years old has amenorrhea for 4 months or more, with two types of serum follicle stimulation hormone (FSH) levels (obtained at least 1 month apart) in the menopausal range. Approximately 2% of women with isolated spontaneous 46, XX POI and 14% with familial spontaneous 46, XX POI are diagnosed with *FMR1* premutation (8). POI occurs in approximately 20% of women who carry the premutation allele in contrast to 1% in the general population, and is designated as FXPOI (2, 9).

Contrary to the risk to develop FXTAS, the relationship between the number of CGG repeats and the risk to develop FXPOI is not linear, with maximum risk confining to carriers with an expansion of 80–100 CGG repeats (10, 11).

Nevertheless, it is now well-documented that both female and male premutation carriers might suffer from associated medical comorbidities beyond FXTAS and FXPOI (12, 13).

Whether Fragile X premutation has a subtle effect on cognition has been under debate for several years. Recent findings point to specific learning disabilities, including significant dyscalculia and ADHD (mainly inattentive subtype). The range of full Fragile X-associated developmental problems, including autism spectrum and attention-deficit/hyperactivity disorder, has been seen with increased frequency in male premutation carriers. In women, if it exists, the symptoms are subtler, and although reported in carrier mothers of Fragile X syndrome children, sometimes the reports refer to women with a full expansion (1).

Unlike the risk of FXPOI being related to a specific range of premutation and less affecting a higher range of premutation and full mutation carrier women, studies found negative linear associations between the repeat length of CGG repeats and verbal IQ, working memory, and letter-number sequencing, as well as a significant negative correlation between repeat length and arithmetic scores (14, 15). Elevated rates of subclinical ASD-related personality and language features among women with the premutation were recently reported, as well as increased self-reported difficulties in executive functioning (16).

In families of Fragile X children, some of the women are revealed as carriers after a child was diagnosed with FXS. As such, mothers of children with FXS are thought to be asymptomatic carriers, while if examined, their genetic results may show the whole range of trinucleotide repeats from premutation to full mutation (1). These mothers may have subtle symptoms that they may not have been aware of or able to connect to Fragile X until the child was diagnosed. In addition to the burden of Fragile X premutation symptomatology, mothers of FXS children and adults face daily challenges when parenting a person with a disability (17). Cognitive characteristics, attention, and executive function in *FMR1* premutation women have been studied and debated; however, it can be assumed that it may influence the life satisfaction and participation of mothers of children with Fragile X (18).

Despite important findings, these studies have many methodological problems, and there is an urgent need for more research examining the phenotype–genotype correlation of *FMR1* premutation (19).

Research on the pathogenesis of brain neurodegenerative processes, which are different from full mutation epigenetic processes, may shed light on common disorders such as intellectual disability, autism spectrum disorder, and ADHD. Nevertheless, the new generation of FXS pharmacotherapy may play a role in the treatment of learning disabilities, FXTAS, and other neuropsychiatric symptoms of permutated individuals.

In addition, if women with *FMR1* premutation do have subtle difficulties influencing functioning and participation, it is extremely important to increase awareness and provide appropriate support to families with Fragile X.

We assume that there is a continuum of learning and attention deficits that correlate with an increased number of repeats. Specific deficits such as right-left disorientation as well as general symptoms of anxiety and attention may influence important daily tasks, such as the ability to drive a car, and result in less participation (20, 21).

## Materials and methods

### Aim of the current study

This study aimed to characterize *FMR1* mothers of children with FXS for the full range of CGG *FMR1* expansion from 55 repeats to more than 200 repeats and to compare function and participation between women having a premutation to women with a full mutation of FMR1 but without showing Fragile X syndrome or the FXTAS phenotype.

### Participants

In total, 108 women were referred to the Keshet Center for autism due to a related FXS patient, mainly offspring or sibling. Participants' age range was 23–77 years old (M = 44, SD = 11.5). Only healthy carrier women were included in the study; women with FXS including overt autism and/or intellectual disability, women with symptoms of FXTAS, and women that reported recent changes in function or behavior were excluded from the study.

### Study design

Data were collected from index cases of FXS patients and relatives from the Fragile X Clinic and National Resource Center at the Keshet Center for autism in Israel. The information included genetic results of CGG repeats, various questionnaires, the presence of learning disabilities in language and/or mathematics, right and left disorientation, and the ability to drive a car (as specified below). The group carrying *FMR1* premutation was analyzed separately and compared to the group carrying a full mutation in the *FMR1* gene.

### Tools and methods

A structured self-reporting telephone questionnaire was administered. Most of the questions were yes–no questions. The questionnaire included several parts: demographics, information regarding fertility, learning abilities including formally diagnosed learning disabilities and ADHD, daily function skills, and participation, as specified in the results section. Participants filled out a general demographic questionnaire including information on occupation, education, and psychosocial familial information including information on the first-degree relatives with FXS.

A history of psychological and psychiatric difficulties, endocrine dysfunction, and fertility in the past and present was retrieved, including the use of medications.

Attention was assessed with regard to formal past or present diagnosis, medication use, and also by rating the Adult ADHD Self-Report Scale Symptom Checklist. Learning was assessed by analyzing years of formal education, history of school assistance and reasons for dropping out if occurred, a reading exercise, and a simple mathematical task.

Daily living skills were assessed in regard to specific abilities such as driving, writing checks, maps and orientation, and organizing tasks and schedules. For example, Do you have difficulty maintaining concentration during activities at work or at home? Do you have difficulty distinguishing between left and right? Do you get confused with dates and times resulting in missed appointments? Clinical traits that may be present in *FMR1* carriers were examined in the context of the impact of daily living skills on participation and function.

The influence of learning and attention difficulties on daily living skills was assessed by specific questions regarding the memory of phone numbers; driving; spatial orientation (right and left confusion); memory of forward and backward month order naming; distractibility in daily tasks; difficulties in reading, keeping a schedule, and writing checks; getting on the wrong bus in reading own handwriting; and difficulties in reading forms and documents, taking and relaying messages, and speaking in public.

It should be noted that the number of answers available on the questionnaire was not always reflective of the full cohort as some women did not provide part of the information. A full table (Table 1) of the tested skills and demographics with the content number of available answers according to group size is reported below.

**Table 1:** Comparisons of full-scale repeats or categorical comparisons (< or ≥200 repeats, NS, non-significant difference) using Pearson's and Wilcoxon tests (Chi-square and Z approximation, respectively).

	**All cohort**	**<200 repeats**	**≥200 repeats**		
**Question**	***N*** **of answers**	***N*** **(%)**	***N*** **(%)**	**Statistic**	**p**
Diagnosed with FXPOI	78	11 (14.10%)	1 (1.28%)		NS
Received psychological/ psychiatric treatment in the past	75	22 (29.33%)	2 (2.67%)		NS
Receives Psychological/ Psychiatric treatment today	77	6 (7.79%)	0 (0%)		NS
Receives psychiatric medication today or in the past	78	17 (21.79%)	5 (6.41%)		NS
Currently suffers from depression or anxiety	78	24 (30.77%)	5 (6.41%)		NS
Attention deficits	78	18 (23.08%)	6 (7.69%)		NS
Difficulty to maintain attention span during activities	78	12 (15.38%)	3 (3.85%)		NS
Easily distracted while doing chores	78	18 (23.08%)	5 (6.41%)		NS
Difficulty in discerning between left and right	78	16 (20.51%)	2 (2.56%)	Z = 1.42	0.15
Difficulties in map reading or spatial orientation in a new place	78	18 (23.08%)	5 (6.41%)		NS
Does not like to read out loud	78	21 (26.92%)	3 (3.85%)		NS
Taking longer to read one page	78	17 (21.79%)	1 (1.28%)		NS
Difficulty in remembering the reading content afterwards	78	21 (26.92%)	3 (3.85%)		NS
Does not like to read long books	78	16 (20.51%)	58 (6.41%)		NS
Makes spelling mistakes	78	9 (11.54%)	2 (2.56%)	Z = 1.54	0.12
Difficulties reading own handwrite	78	8 (10.26%)	1 (1.28%)		NS
Gets confused speaking in front of an audience	78	24 (30.77%)	5 (6.41%)		NS
Difficulties taking phone messages and delivering them	78	8 (10.26%)	1 (1.28%)		NS
Difficulty pronouncing a longer word correctly	78	13 (16.67%)	4 (5.13%)	Z = 1.69	0.09
Math mental addition exercise	78	14 (17.95%)	2 (2.56%)		NS
Did not answer		2 (4.76%)	0.00%	Z = −1.65	0.1
Took a few seconds but answered		14 (33.33%)	3 (7.14%)		
Answered quickly		16 (38.1%)	3 (7.14%)		
Got it wrong		2 (4.76%)	2 (4.76%)		
Tends to confuse punching numbers on the phone	78	1 (1.28%)	0 (0%)		NS
Difficulty naming the months of the year in order	78	2 (2.56%)	0 (0%)		NS
Difficulty naming the months in reverse order	77	10 (12.99%)	1 (1.30%)		NS
Confuses times and schedules	78	10 (12.82%)	0 (0%)		NS
Frequently makes mistakes in writing checks	78	5 (6.41%)	0 (0%)		NS
Thinks that documents and forms are confusing and hard to understand	78	17 (21.79%)	3 (3.85%)		NS
Confuses bus number lines (e.g., 95 and 59)	78	4 (5.13%)	0 (0%)		NS
Had difficulties learning the multiplication table in school	78	17 (21.79%)	2 (2.56%)		NS
Diagnosed with ADHD or other learning disability in the past	78	3 (3.85%)	3 (3.85%)	Chi^2^ = 5.2	0.02
Got adaptations during school years	55	5 (9.09%)	2 (3.64%)		NS
Started studying but did not finish	78	20 (25.64%)	1 (1.28%)		NS
Does not hold driver's license	78	12 (15.38%)	7 (8.97%)	Z = −2.22	0.026
Does not drive	78	14 (17.95%)	8 (10.26%)	Z = −2.53	0.01

### Statistical analysis

Analysis was performed using the Pearson correlation test, and for analyzing differences between groups in each pair with non-parametric comparisons, the Wilcoxon (mean scores difference) test was used.

The participants' number of CGG repetitions in the different groups was investigated in three ways: as an independent nominal factor dividing participants' repetitions into two groups of <200 and ≥200 repetitions and testing its significance with the questionnaire responses. To complement the analysis, we also presented the number of repetitions as a continuous dependent factor of the questionnaire's results. Third, a subset within the <200 repetitions was analyzed as a dependent variable.

Our rationale in choosing to present our various analyses with the number of CGG repetitions both as an explaining (independent) variable and a dependent variable, CGG repeats should be tested as the “driver” of some low daily skill functioning by using binomial comparisons (Pearson's test) or multinomial regression models with the number of repetitions as the independent variable. However, by using the number of repetitions as the dependent variable, we can further support and suggest that CGG repetitions have an association and correlate with daily functions within the tested subgroups.

## Results

### Demographics

From 150 women who gave consent, data were analyzed for 108 women with valid genetic data (42 without specification of the number of CGG repeats were excluded). The age of the cohort ranged from 23 to 77 (at the time of the questionnaire, M = 44, SD = 11.5).

A total of nine women (8.6%) reported that they have never heard of premutation complications such as FXTAS. In total, 20 women were reported to be treated medically for hypothyroidism, type 2 diabetes, hypertension, hypercholesterolemia, and medications for anxiety or depression (see Table 1 for the rates of psychiatric complaints).

### Marital status

Of the total respondents, 62 were married (59%), 14 were divorced (13.3%), five were single (4.8%), one was widowed, and 1 was in a relationship. Socioeconomic status: Among the participants, 24 belonged to the below-average category (22.9%), 28 belonged to the average category (26.7%), and 28 belonged to the above-average category (26.7%).

A total of 101 women were mothers, and five mothers did not have children (two mothers did not have fragile information). In total, the women in our sample had 307 children between 1 and 7 children per woman, or an average of 3.2 children. In total, 81 mothers had healthy children (a total of 175 healthy children, with an average of 1.8 children); Eighty-eight of the women had children with FXS, having a total of 122 children with FXS, with an average of 1.3 children (10 children had no information).

### Genetic results

The number of CGG repeats was 55–700 (M = 138.2, SD = 111.5). Most women (n = 97, 82.9%) did not perform the genetic testing during pregnancy or before and performed it after the child with FXS was born and diagnosed. The number of children with FXS for each woman was between 0 and 6, with an overall number of children between 0 and 8.

A total of 84 women carried the premutation of 56–199 CGG repeats, and 24 women carried a full mutation of more than 200 CGG repeats on the *FMR1* gene, without meeting the criteria for FXS diagnosis.

### Fertility and obstetrics information

A total of 78 women (57.4%) had a natural birth, 14 (20.6%) had a cesarian section; in 10 (22.1%) women, a vacuum-assisted vaginal extraction was used. In total, 114 women (97.4%) had spontaneous pregnancy, and in 3 (2.6%), there was the use of IUI, medications, or IVF.

When analyzed as a continuous variable within the <200 CGG repeats group, there was a significant increase in reported C-section deliveries that correlated with a higher number of repeats. Using the Wilcoxon method for each paired comparison, we observe that a higher number of repeats is significantly associated with C-section deliveries, i.e., natural birth, N = 47; C-section, N = 5; vacuum, N = 7, where C-section appears to be more significant than vacuum and natural deliveries; Z (C-section to natural birth) = 1.57, *p* = 0.11 (see Table 2).

**Table 2:** Comparisons within the <200 group.

**Question**	**N of < 200 repeats**	**Statistic**	**Significance**
Difficulty in discerning between left and right	16	Z = 2.25	0.025
No Difficulty in remembering reading contents afterwards	44	Z = 1.67	0.1
Makes spelling mistakes	9	Z = 1.91	0.056
**A simple math exercise**			
Did not answer	2	Z = −1.62	0.1
Took a few seconds but answered	14	Z = −2.04	0.04
Answered quickly	16		
Got it wrong	2		
Confuses times and schedules	10	Z = 2.92	0.0035
Frequently makes mistakes in writing bank checks	5	Z = 1.79	0.074

### Education

**Figure 1:**
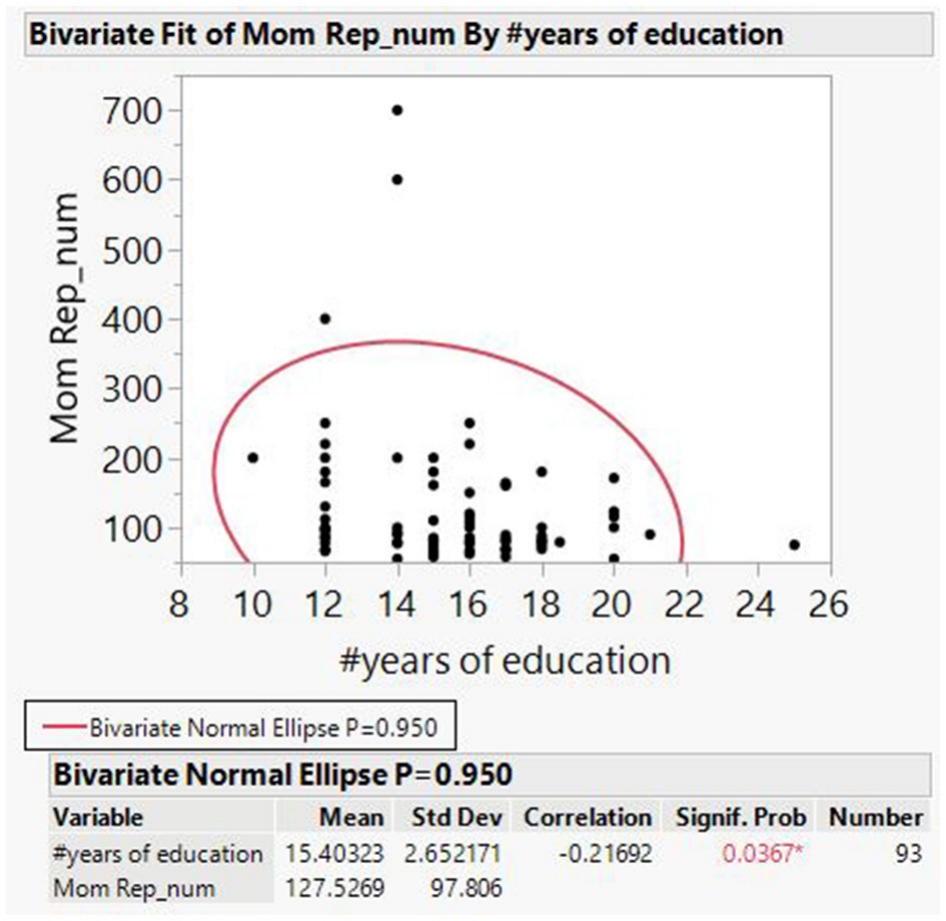
Bivariate fit of number of CGG repetitions by years of education.

Women had a range of 10–25 years of education) M = 15.4, SD = 2.7), and 23% of the women (N = 23) started but did not complete their studies. Fewer years of education significantly correlated with higher CGG repeats albeit low fit (bivariate normal ellipse R = −0.22, *p* < 0.05, Figure 1).

### Attention and learning

Attention and learning were examined by questionnaires and by directly administered exercises of simple mathematical tasks and reading and memory tasks:

A rather simple mathematical exercise that requires some calculation of numbers was tested. Groups within this test were “did not answer at all,” N = 2; “took a few seconds to answer,” N = 14; “answered quickly,” N = 16; and “got it wrong,” N = 2. Within the <200 CGG repeat group, women with a higher number of repetitions showed a longer time to answer or did not answer it at all as compared with those that answered quickly (Z (took some time over answered quickly) = −2.04, p < 0.05; Z (did not answer over answered quickly) = −1.62 *p* = 0.1). The same observation was repeated in the >200 categorical comparisons (Z = −1.65, *p* = 0.1).

Women within the <200 group and with a higher number of repetitions had a stronger tendency to have spelling mistakes (“no spelling mistakes,” N = 67; “had spelling mistakes,” N = 9; Z = 1.91, *p* = 0.056). Women in the <200 group with higher CGG repeats had a higher tendency to make mistakes while writing checks (N = 5) (Wilcoxon Z = 1.79, *p* = 0.074) (see Table 2).

Women within the wider group of CGG repeats (both <200 and ≥200, N = 17) also had difficulties pronouncing a longer word correctly (Wilcoxon Z = 1.69, *p* = 0.09).

Women within the <200 group had difficulties discerning left from right (N = 16) (Wilcoxon Z = 2.25, *p* < 0.05); they also had difficulties in remembering a reading content when asked about it a few moments afterward (Wilcoxon Z = 1.67, *p* = 0.1).

When asked whether or not they had a diagnosis of ADHD or other learning disabilities in the past, women in the >200 group were associated with a positive answer (Pearson's chi-square = 5.2, *p* = 0.02).

### Daily function skills

#### Driving experience

Performing a Wilcoxon test when testing for differences in the CGG repeats means that women without a driver's license tended to appear more among participants with higher CGG repeats (Z = −2.22, *p* < 0.05). Furthermore, of those holding a driver's license, we observed that women who refrain from driving have higher CGG repeats on average than women who actually drive (Z = −2.53, *p* < 0.05).

All the other tested parameters showed no significance in women with a higher number of repetitions. However, none of the lower number of repeats showed any significance to the tested skills, further supporting the assumption that difficulties in daily skills were more strongly associated with a higher number of repeats.

## Discussion

There is growing evidence that women with premutation face medical and emotional challenges during their lives beyond the risk of bearing a child with FXS. Prior studies showed that premutation carriers have an increased risk of infertility as linked to FXPOI, learning disabilities, neuropsychiatric issues, ADHD, and endocrine dysfunction. Some of the impairments—mainly fertility issues, were described as premutation carrier features only and were not described in female carriers of the full mutation. As is FXTAS, our studies support the findings that specific learning disabilities are phenotypic features of the higher end of CGG repetitions including female carriers of full mutations and are not characteristic of premutation carriers of a lower number of repeats.

The metabotropic glutamate receptor (mGluR) hypothesis states that the neurological deficits in individuals with FXS are mainly due to downstream consequences of overstimulation of the mGluR pathway. Deficits in the GABA receptors in different regions of the brain are associated with behavioral and attentional processing deficits linked to anxiety and autistic behaviors (22). We postulated that the neuropsychiatric comorbidities can be associated with an increased number of CGG repeats and that the severity increases as a continuum between premutation and full mutation of *FMR1*. Our pilot epidemiologic evaluations of the carriers of premutation and full mutation resulted in multiple symptomatologies: specific learning disabilities (mainly dyscalculia), right and left confusion, and attention deficits.

A recent study documented CGG repeat count and stress that independently contributed to executive dysfunction in female *FMR1* premutation carriers (23). As in our study, a higher level of education was emphasized as a “protective factor” (24).

Another recent study found three clusters of *FMR1* premutation carriers: Cluster 1 represented a psychiatric feature group; cluster 2 represented a group with executive dysfunction and elevated high-frequency neural oscillatory activity; and cluster 3 represented a relatively unaffected group. Among women in cluster 1, a higher CGG repeat count was significantly related to more severe distractibility. Cluster 2 was defined by executive dysfunction and an electrophysiological profile of reduced theta and increased gamma 1 and gamma 2 waves (25).

While difficulties in some daily living skills show significance toward a higher number of CGG repeats, a similar tendency was not observed for a lower number of CGG, further strengthening our hypothesis of the difficulties faced by mothers of children with FXS.

Math skills and dyscalculia may relate to right and left confusion, as described in the developmental entity of “developmental Gerstman Syndrome.” This term was coined by Kinsbourne in 1963 and linked to learning disabilities ever since (26). The entity is theoretical since the overt anatomical abnormalities near the angular gyrus of the dominant hemisphere are absent in the developmental entity (27). This association may affect the function and influence spatial orientation, driving abilities, and reading a map. Attention and reading skills may influence remembering phone numbers and messages, organizing the calendar and daily tasks.

The encouraging findings are that despite evidence of learning and attention difficulties, most carriers of premutation and full mutation women function well in most daily skills. These findings are consistent with several studies that found that the neuropsychiatric functioning of female *FMR1* premutation carriers was relatively similar to matched controls (12, 28). Nevertheless, they face significant difficulties in specific areas of functioning such as driving, and confusion in times and schedules. Those daily function skills are mostly impacted by dyscalculia, right and left disorientation, and attention-related difficulties. This conclusion may aid to design specific interventions to address education and skill development in order to improve the daily functioning of premutation carriers.

Our study supports prior findings that the premutation allele may lead to other disorders in addition to FXTAS and FXPOI. Some attention and learning difficulties correlate with an increased number of CGG repeats and are in fact prevalent features of premutation in women. Those difficulties may interfere with the performance of daily tasks, but even minor difficulties should be emphasized and addressed, especially in the view that many carrier mothers also care for children with disabilities.

### Limitations

The study used mainly self-report questionnaires. Participants were asked whether a diagnosis of ADHD or other learning disabilities has been received and about their subjective perception of difficulties, and no additional formal diagnosis was made for ADHD or for learning disabilities in the whole cohort. Detailed examinations including ADHD, executive function tasks, mathematics, and verbal exercises were performed in smaller subgroups of premutation carriers, and the examination results correlated well with the questionnaire's self-reported answers. Specific data were available only from a limited number of participants, and as such, some findings did not reach significance. This study should be replicated in a larger cohort. Another limitation is that we refer to the number of CGG repeats, but clearly, the genetic and epigenetic mechanisms are much more complicated. When discussing female carriers of premutation, we have to take into consideration the process of X inactivation. We did not check in our study the percentage of inactivation. Nevertheless, the peripheral blood measurements may not be representative of activation in the brain tissue. As such, due to the large sample size and assuming random inactivation in most cases, we considered a CGG number as being a rough representation of the mutation burden (29).

### Implications

This study has focused on the various areas of abilities, skills, and life participation of Fragile X premutation and full mutation women. The specific profile of learning difficulties found in the study has a direct impact on daily living skills, such as driving. This is aimed at finding areas that need to be addressed in order to improve their quality of life, skills, and participation.

## Data availability statement

The raw data supporting the conclusions of this article will be made available by the authors, without undue reservation.

## Ethics statement

The study has been approved by the appropriate Institutional Research Ethics Committee “Sheba Medical Center” Institution Review Board - Helsinki approval number 9187-11-SMC. The patients/participants provided their written informed consent to participate in this study.

## Author contributions

LG designed the study and the data collection instruments, contributed to conception and design of the study, and wrote the first draft of the manuscript. MS performed the statistical analysis and wrote sections of the manuscript. OA coordinated and supervised data collection and wrote sections of the manuscript. TK, SD, YB-L, and DM-T collected the data, carried out initial analyses, and organized the database. SS was in charge of the ethical approvals of the study and of data management. DG contributed to literature update and reviewed and critically revised the manuscript. MA, OB, and OS contributed to conception and design of the study, supervised data analysis, and critically revised manuscript. All authors contributed to manuscript revision and approved the submitted final version of the manuscript.
